# BRCA1-mutated and basal-like breast cancers have similar aCGH profiles and a high incidence of protein truncating TP53 mutations

**DOI:** 10.1186/1471-2407-10-654

**Published:** 2010-11-30

**Authors:** Henne Holstege, Hugo M Horlings, Arno Velds, Anita Langerød, Anne-Lise Børresen-Dale, Marc J van de Vijver, Petra M Nederlof, Jos Jonkers

**Affiliations:** 1 Division of Molecular Biology, Netherlands Cancer Institute, Plesmanlaan 121, 1066 CX Amsterdam, The Netherlands; 2 Department of Pathology, Netherlands Cancer Institute, Plesmanlaan 121, 1066 CX Amsterdam, The Netherlands; 3 Central Microarray Facility, Netherlands Cancer Institute, Plesmanlaan 121, 1066 CX Amsterdam, The Netherlands; 4 Department of Genetics, Institute for Cancer Research, Norwegian Radium Hospital, Oslo University Hospital, Montebello, NO-0310 Oslo, Norway; 5 Department of Pathology, Academic Medical Center, Meibergdreef 9, 1105 AZ Amsterdam, the Netherlands

## Abstract

**Background:**

Basal-like breast cancers (BLBC) are aggressive breast cancers for which, so far, no targeted therapy is available because they typically lack expression of hormone receptors and HER2. Phenotypic features of BLBCs, such as clinical presentation and early age of onset, resemble those of breast tumors from *BRCA1*-mutation carriers. The genomic instability of *BRCA1*-mutated tumors can be effectively targeted with DNA-damaging agents and poly-(ADP-ribose) polymerase 1 (PARP1) inhibitors. Molecular similarities between BLBCs and *BRCA1*-mutated tumors may therefore provide predictive markers for therapeutic response of BLBCs.

**Methods:**

There are several known molecular features characteristic for *BRCA1*-mutated breast tumors: 1) increased numbers of genomic aberrations, 2) a distinct pattern of genomic aberrations, 3) a high frequency of *TP53 *mutations and 4) a high incidence of complex, protein-truncating *TP53 *mutations. We compared the frequency of *TP53 *mutations and the pattern and amount of genomic aberrations between *BRCA1*-mutated breast tumors, BLBCs and luminal breast tumors by *TP53 *gene sequencing and array-based comparative genomics hybridization (aCGH) analysis.

**Results:**

We found that the high incidence of protein truncating *TP53 *mutations and the pattern and amount of genomic aberrations specific for BRCA1-mutated breast tumors are also characteristic for BLBCs and different from luminal breast tumors.

**Conclusions:**

Complex, protein truncating TP53 mutations in BRCA1-mutated tumors may be a direct consequence of genomic instability caused by BRCA1 loss, therefore, the presence of these types of TP53 mutations in sporadic BLBCs might be a hallmark of BRCAness and a potential biomarker for sensitivity to PARP inhibition. Also, our data suggest that a small subset of genomic regions may be used to identify BRCA1-like BLBCs. BLBCs share molecular features that were previously found to be specific for BRCA1-mutated breast tumors. These features might be useful for the identification of tumors with increased sensitivity to (high-dose or dose-dense) alkylating agents and PARP inhibitors.

## Background

Lobules and ducts within the normal human breast are lined with a double layer of epithelial cells: an inner layer of luminal cells and an outer layer of basal/myoepithelial cells that are in direct contact with the basement membrane. Transformation of different mammary epithelial cells results in considerable heterogeneity in breast cancer subtypes, giving rise to differences in clinical presentation, histology and response to therapy [1]. Gene expression profiling has identified five molecular breast tumor subtypes: luminal A, luminal B, normal breast-like, human epidermal growth factor 2 (HER2/ERBB2) positive, and basal-like [2,3]. In the clinic however, only immunohistochemistry data for estrogen receptor (ER), progesterone receptor (PR) and HER2 status are used to guide treatment choice [4].

Approximately ~70-80% of all breast tumors are hormone receptor positive [5] and therefore sensitive to adjuvant endocrine therapy [6]. These tumors classify mostly as luminal A/B breast tumors [3]. The addition of trastuzumab to adjuvant chemotherapy considerably improved the outcome of HER2-positive breast tumors [7]. However, approximately 10-20% of all breast tumors do not express hormone receptors or HER2, and are therefore insensitive to endocrine or trastuzumab treatment. Currently, the only treatment available for these triple-negative breast cancers (TNBCs) is cytotoxic chemotherapy [8].

The TNBC group as defined by immunohistochemical staining consists for approximately 80% of basal-like breast cancers (BLBCs) as defined by gene expression profiling [9]. BLBCs express luminal (CK19 and CK18) as well as basal cytokeratins (CK5/6, CK17 and CK14), suggesting that these tumors originate from an undifferentiated, dual-lineage stem/progenitor cell type. Furthermore, the *TP53 *gene is often mutated in BLBCs [10], and the gene expression profiles of *TP53*-mutated breast tumors show strong association with the BLBC subgroup [11]. Consequently, BLBCs are aggressive tumors with an expansive growth pattern (pushing margins), a high proliferation rate, high relapse rates and poor survival. Moreover, BLBCs occur more frequently in premenopausal women than in postmenopausal women and are often difficult to detect by mammography or ultrasound [9].

Although TNBC/BLBC has a relatively poor prognosis in the first five years after diagnosis, approximately 60% of patients - even without adjuvant chemotherapy - do *not *relapse, and after ~8 years of follow up have a high chance of being cured (reviewed in [9]). This indicates that within the TNBC/BLBC tumor group there is considerable heterogeneity in tumor behavior. At present however, all TNBC/BLBC patients are treated with cytotoxic adjuvant chemotherapy because there are no clinically useful prognostic and predictive markers to identify patients with aggressive, chemotherapy-sensitive TNBC/BLBC, leading to unnecessary exposure to chemotherapy of a substantial number of patients [8]. In recent years it is becoming clear that phenotypic features of TNBC/BLBC may also apply to the majority of hereditary *BRCA1*-mutated breast tumors [12,13]. Since BRCA1 function is required for homology-directed repair of DNA double-strand breaks (DSBs), *BRCA1*-mutated tumors and BRCA1-like BLBCs are predicted to be sensitive to DSB inducing therapy [14]. Indeed, breast tumors from *BRCA1*-mutation carriers are sensitive to inhibition of DNA single-strand break (SSB) repair by poly(ADP-ribose) polymerase (PARP) inhibitors [15] and to chemotherapy that causes DSBs, such as platinum drugs, alkylating agents and topoisomerase I poisons [16,17]. It will therefore be important to identify features of sporadic BLBCs [14] that may be useful as predictive biomarkers for response to DSB-inducing chemotherapy or PARP inhibitors. Known molecular features characteristic for *BRCA1*-mutated breast tumors are 1) a high degree of genomic instability due to homologous recombination (HR) deficiency [18], 2) a distinct pattern of genomic aberrations [19-22] 3) a high frequency of *TP53 *mutations and 4) a high incidence of complex, protein-truncating *TP53 *mutations [10,23]. In this study, we determined to what extent these molecular characteristics of *BRCA1*-mutated tumors are present in BLBCs.

## Methods

### Breast tumor groups

To compare molecular characteristics of *BRCA1*-mutated tumors with BLBCs, we used data from two published tumor sets from the Netherlands Cancer Institute that were sequenced for *TP53 *and for which aCGH data was generated. The first dataset contains 27 *BRCA1*-mutated breast tumors and 17 luminal breast tumors (defined by expression profiling) that were previously described by Joosse *et al *[24]. The luminal tumors from this study were designated luminal-J: J for *Joosse*. The second dataset from Horlings *et al.*, [25] contained 21 non-hereditary BLBCs and 31 luminal breast tumors that were part of a series of 295 breast tumor specimens [26] which were assigned to breast cancer subgroups according tot their gene expression profiles [2,3]. For privacy reasons *BRCA1*-mutation status was not verified in the BLBCs, however, these patients did not have a family history of breast cancer. Luminal tumors from this study were labeled luminal-H, H for *Horlings*. Six luminal breast tumors were used in both the Joosse study and the Horlings study. *TP53 *mutation, ER/PR/HER2, CK56 staining pattern and age at diagnosis of all tumors are shown in Table 1.

**Table 1:** Tumor characteristics of BRCA1-related, Luminal-J, basal-like and Luminal-H breast tumors.

NKI ID	deleterious TP53 mutation by prediction	complex/truncatingTP53 mutation	hotspot mutation	TP53 IHC	ER IHC	PR IHC	HER2 IHC	CK5/6IHC	breast cancer subtype/BRCA1 mutation	Age at diagnosis
**BRCA1-mutated breast tumors**										
B107	G266E	0	1	0	0	0	0	NA	c.1319delT	41
B109	**R213X**, H214Y	1	1	0	0	0	0	NA	c.IVS21-36del510	30
B116	Y163C	0	1	1	0	0	0	NA	c.185delAG	49
B118	NA	NA	NA	0	0	0	0	NA	c.4416_4417delTTinsG	34
B119	NA	NA	NA	0	0	0	0	NA	c.4416_4417delTTinsG	34
B122	**239 insT**	1	0	0	0	0	0	NA	c.4446C > T	45
B124	NA	NA	NA	1	0	0	0	NA	c.3875del4	61
B125	NA	NA	NA	0	0	0	0	NA	c.2804delAA	35
B126	**del 255**	1	0	1	0	0	0	NA	c.IVS21-36del510	40
B127	*T55I*	0	0	1	0	0	0	NA	c.5382insC	39
B135	wild type	0	0	1	0	0	0	NA	c.2312del5	41
B137	**224 splice G > A**	1	0	0	0	0	0	NA	c.IVS12-1632del3835	32
B141	wild type	0	0	0	0	0	0	NA	c.IVS21-36del510	39
B145	**110 delC, Q100X**	1	0	0	0	0	0	NA	c.185delAG	33
B146	**145 delG, Q104X**, *P98S*	1	0	0	0	0	0	NA	c.185delAG	33
B149	R273H	0	1	1	0	0	0	NA	c.IVS20+1G > A	31
B150	V216 M, *P223 S, R290C*	0	1	0	0	0	0	NA	c.IVS21-36del510	41
B152	R175H	0	1	1	0	0	0	NA	c.IVS13+4123ins6081	47
B153	**del 155-156**	1	0	1	0	0	0	NA	c.185delAG	48
B156	R248W, R280K, *V218I*	0	1	1	0	0	0	NA	c.5382insC	47
B158	R282G, E326K	0	1	1	0	0	0	NA	c.IVS21-36del510	27
B160	**167 insA**	1	0	0	0	0	0	NA	c.IVS20+1G > A	61
B161	**R213X**, R282W, P151R	1	1	1	0	0	0	NA	c.IVS13+4123ins6081	30
B162	NA	NA	NA	0	0	0	0	NA	c.IVS20+1G > A	27
B164	**258 delG**	1	0	0	0	0	0	NA	c.del exons 1A-7	31
B165	NA	NA	NA	1	0	0	0	NA	c.IVS12-1632del3835	33
B171	**R306X**	1	0	0	0	0	1	NA	c.IVS2-9C > G	33

**Luminal-J breast tumors**										
C002	R248W, R110C, *T55I*	0	1	0	0	0	1	0	Luminal A	45
C010	NA	NA	NA	0	1	1	0	0	Luminal A	40
C017	**K305X**	1	0	0	1	1	0	0	Luminal B	36
C020	wild type	0	0	1	0	0	0	1	Luminal A	34
C022	NA	NA	NA	0	1	0	0	0	Luminal B	51
C025	P177L	0	1	0	1	1	0	0	Luminal A	50
C027	NA	NA	NA	0	1	1	1	0	Luminal A	45
C028	wild type	0	0	0	1	1	0	0	Luminal B	41
C030	wild type	0	0	0	1	0	0	0	Luminal B	38
C034	wild type	0	0	0	1	1	0	0	Luminal A	47
C036	wild type	0	0	0	1	1	0	0	Luminal A	40
C037	NA	NA	NA	0	1	1	0	0	Luminal B	49
C042	*P190L*	0	0	0	1	1	0	0	Luminal B	42
C044	wild type	0	0	0	1	0	0	0	Luminal B	45
C052	wild type	0	0	0	1	1	0	0	Luminal A	45
C057	*P98L*	0	0	0	0	0	0	0	Luminal B	51
C060	H179R, *T125M*	0	1	1	1	1	0	0	Luminal B	47

**Basal-like breast tumors**										
131	**239_240delCA**	1	0	1	0	0	0	1	Basal-like	39
135	C242Y	0	1	1	0	0	0	1	Basal-like	45
164	**W53X**	1	0	0	0	0	0	1	Basal-like	50
184	**183_184insC**	1	0	0	0	0	0	1	Basal-like	44
215	**110delC**	1	0	0	0	0	0	1	Basal-like	49
228	**IVS5-2 A > C (splice)**	1	0	0	0	0	0	0	Basal-like	39
230	**E221X**	1	0	0	0	0	1	0	Basal-like	28
238	V173M	0	1	1	0	0	0	1	Basal-like	42
241	**R196X**	1	0	0	0	0	0	1	Basal-like	41
268	*L252P*	0	0	1	0	0	0	1	Basal-like	38
269	*N131S*	0	0	0	0	1	0	1	Basal-like	38
270	**283insGC**	1	0	0	0	0	0	1	Basal-like	50
307	**218delGTG**	1	0	1	0	0	0	1	Basal-like	44
324	I195T	0	1	1	0	0	0	1	Basal-like	46
326	**155_156del**	1	0	1	0	0	0	1	Basal-like	39
330	**201delT**	1	0	0	0	0	0	1	Basal-like	26
332	Q286K	0	1	1	0	0	0	1	Basal-like	49
335	R248W	0	1	1	0	0	0	1	Basal-like	48
367	wild type	0	0	1	1	0	1	0	Basal-like	49
377	**255_256delTCA**	1	0	1	1	1	0	1	Basal-like	52
398	Y220C	0	1	1	0	0	0	1	Basal-like	34

**Luminal-H breast tumors**										
6	wild type	0	0	0	1	1	0	0	Luminal A	49
107	**Q317X**	1	0	0	1	1	0	NA	Luminal B	38
110	wild type	0	0	0	1	1	0	0	Luminal B	51
145	R273C	0	1	0	0	1	0	0	Luminal B	48
157 (C002)*	R248W	0	1	0	0	0	1	0	Luminal A	45
166	wild type	0	0	0	1	0	0	0	Luminal B	43
167	wild type	0	0	0	1	1	1	0	Luminal A	44
176	wild type	0	0	0	0	1	0	0	Luminal A	46
203	wild type	0	0	0	1	1	1	0	Luminal A	49
205	K132R	0	1	0	1	1	0	1	Luminal A	50
214	wild type	0	0	0	1	1	0	0	Luminal A	41
220	wild type	0	0	0	1	1	0	0	Luminal A	42
231	wild type	0	0	0	1	1	NA	0	Luminal A	43
240 (C060)*	H179R	0	1	0	1	1	0	0	Luminal B	47
295	wild type	0	0	0	1	1	0	0	Luminal A	48
298	wild type	0	0	1	1	1	0	0	Luminal A	50
302 (C034)*	wild type	0	0	0	1	1	0	0	Luminal A	47
305 (C036)*	wild type	0	0	0	1	1	0	0	Luminal A	40
312	**205delT**	1	0	0	1	1	0	0	Luminal B	47
318	R110L, S127C	0	1	0	1	1	0	0	Luminal A	37
322 (C044)*	wild type	0	0	0	1	0	0	0	Luminal B	45
329	wild type	0	0	0	1	1	0	0	Luminal B	49
346	wild type	0	0	0	1	1	0	0	Luminal A	49
354	P177R	0	1	1	1	1	0	1	Luminal A	47
356	wild type	0	0	0	1	1	0	0	Luminal A	49
361	wild type	0	0	0	1	1	0	0	Luminal A	42
371	wild type	0	0	1	1	0	0	0	Luminal A	51
378	wild type	0	0	0	1	0	0	0	Luminal B	52
389 (C057)*	wild type	0	0	0	1	0	0	0	Luminal B	51
391	wild type	0	0	0	1	0	0	0	Luminal A	51
393	wild type	0	0	0	1	1	0	0	Luminal B	51

TP53 mutations Bold: predicted truncating TP53 mutation (frameshift, splice, nonsense and in-frame insertions or deletions), plain text: hotspot mutations according to Walker *et al*. Italics, missense TP53 mutations predicted deleterious by the SIFT or EffectGroup3 algorithms. Immunohistochemistry data for TP53, ER, PR, HER2 and CK5/6; *BRCA1*-mutation; tumor type as determined by expression profiling; age at diagnosis.

*The TP53 gene of the BRCA1-mutated, Luminal-J tumors were sequenced in a different laboratory than the BLBCs and the luminal-H tumors. Of the 6 overlapping tumors between Luminal-H and Luminal-J tumors a discrepancy occurs in 3 tumors: In luminal-H tumor 157 a R248W mutation is found and in corresponding Luminal-J tumor C002 an additional R110C and T55I mutations are found. In luminal-J tumor C057 a P98L mutation is found and corresponding luminal-H tumor 389 no mutations are found. In luminal-H tumor 240 a H179R mutation is detected and in corresponding luminal-J tumor C060 an additional T125 M mutation is found.

### TP53 mutation analyses

For 21 *BRCA1*-mutated tumors, and 13 luminal-J tumors exons 2-9 of *TP53 *were previously sequenced [23]. The abundance of the aberrant base was estimated from the sequence chromatogram from both the forward and reverse sequencing runs. When comparing mutation types found in the tumor groups, the influence of tumor heterogeneity was minimized by only including *TP53 *mutations that had an estimated abundance of >25% in the tumor DNA [23].

For all BLBC and luminal-H tumors *TP53 *exons 2-11 (including +/- 30 bp outside each exon) were sequenced using AB 3730 DNA Analyzer (reference sequence NM_000546).

Frameshift, splice and nonsense mutations and in-frame insertions/deletions are defined as "complex *TP53 *mutations". All missense mutations found in the *BRCA1*-mutated and luminal-J tumor groups were classified according to their predicted effect on p53 function as determined by the Sorting Intolerant from Tolerant algorithm SIFT; [27,28], as used in the IARC TP53 database [29,30]. Because no matched normal/germ-line DNA was available, some benign germ-line variants may have been identified as deleterious somatic mutations by SIFT. All *TP53 *missense mutations found in the BLBC and luminal-H tumor groups were classified to be deleterious or non-deleterious according to their predicted effect on TP53 function using "EffectGroup3" [31] as used in the IARC TP53 database. The 29 most common hotspot mutations (P < 0.001) identified by Walker and colleagues [32] are referred to as ''hotpot mutations'': K132, C135, P151, V157, R158, Y163, V173, R175, C176, H179, H193, Y205, Y220, Y234, M237, C238, S241, C242, G245, M246, R248, R249, G266, R273, P278, R280, D281, R282, and E285.

### Comparisons aCGH data derived from FFPE and fresh frozen tissue

For aCGH procedures of the *BRCA1*-mutated and luminal-J tumor groups: see Joosse *et al.*, 2009 [24]. For aCGH procedures of the BLBC and luminal-H tumor groups: see Horlings *et al.*, [25]. For the aCGH analyses, a microarray platform containing 3,500 human BAC/PAC clones covering the whole genome with an average spacing of 1 Mb was used [33]. Although the 1 Mb resolution of the BAC platform limits sensitivity for focal changes, the aCGH data is a sound representation of our tumor groups and can be used to find recurrent differences between tumor groups.

When comparing aCGH profiles of tumor DNA isolated from formalin-fixed paraffin-embedded (FFPE) tissue [24] and fresh-frozen tissue [25], we noticed that the log2-ratios obtained from the different DNA sources showed different distributions. From the six overlapping samples between the luminal-H and luminal-J tumor groups, as shown in Table 1 we saw that distribution of log2-ratios derived from FFPE samples was consistently wider than the log2-ratios derived from fresh frozen tissue (Additional File 1). Therefore, we transformed both the FFPE (i.e. all log2 ratios from *BRCA1*-mutated and luminal-J tumors taken together) and the fresh frozen datasets (i.e. all log2 ratios from BLBC and luminal-H tumors taken together) to have a mean of zero and a standard deviation of one before applying KC-SMART and *comparative*-KC-SMART. This enabled us to construct one luminal tumor group consisting of tumors from both the luminal-H and luminal-J tumor groups to compare the *BRCA1*-mutated and BLBC-data with. The six tumors that overlapped between the two groups were included only once and taken from the luminal-J tumor group.

### KC-SMART and comparative-KC-SMART analysis

#### KC-SMART

(Kernel Convolution - a Statistical Method for Aberrant Region detection) is a computational approach for statistical analysis of non-discretized aCGH data from multiple experiments, and determines which regions are significantly gained or lost relative to randomized data (P < 0.05) [34]. We used KC-SMART to smooth the raw log2-ratios by generating a Kernel Smoothed Estimate (KSE) curves for gains (KSE_gains_) and losses (KSE_losses_) separately *across a group *of tumors.

#### Comparative-KC-SMART

[35] detects genomic regions that have a differential aCGH signal *between two tumor groups*. The *comparative*-KC-SMART algorithm smoothes raw log2-ratio data from each individual tumor profile by placing Gaussian kernel functions with the height of the log2-ratio at the genomic midposition of each probe (without separating gains and losses, as done for KC-SMART). For each tumor, an aggregated profile is determined by convolution of locally weighted kernel functions. For each genomic position, the KSE values from all tumors that belong to the two tumor groups in the comparison are used to calculate a signal to noise ratio (SNR). We determined a cut-off that defines significant SNR values by generating SNR data using 6000 class-label permutations and calculating the significance threshold corresponding with a False Discovery Rate (FDR) of 0.05. The width of a kernel applied to each data point determines the extent of smoothing and the size of aberrations detected. Smoothing individual tumors with a kernel width of 20 Mb resulted in aCGH profiles that recapitulated raw aCGH data well; therefore, we chose to use this kernel width for all comparisons. R-packages of KC-SMART and *comparative*-KC-SMART have been submitted to Bioconductor [36]. We used NCBI Build 36 (Hg 18) for these analyses.

### Clustering analysis

We used the MeV program [37] to cluster tumor aCGH data. Samples and genes are hierarchically clustered using pearson correlation and complete linkage with leaf ordening.

## Results

### TP53 mutations in BRCA1-mutated, BLBC and luminal breast cancers

We previously found that nearly all *BRCA1*-mutated breast tumors had deleterious *TP53 *mutations due to an increased frequency of truncating frameshift, splice, nonsense mutations and in-frame insertions and deletions [23]. *BRCA1*-mutated tumors and BLBCs are both basal like TNBCs, characteristics that are different from hormone receptor positive luminal breast tumors (Table 1). Therefore, we were motivated to compare *TP53 *mutation type and frequency found in non-hereditary BLBCs, in *BRCA1*-mutated tumors and in luminal breast tumors. *TP53 *mutation types and other tumor characteristics are listed in Table 1. The *TP53 *gene from the *BRCA1*-mutated/luminal-J tumors and the BLBC/luminal-H tumors were sequenced in different labs with slightly different methods (see Methods section). At the cost of reducing the power of this analysis we wanted to make sure we did not introduce a methodical bias in our comparisons, therefore, the *TP53 *mutation data for luminal-J and luminal-H tumors were not combined.

**Figure 1:**
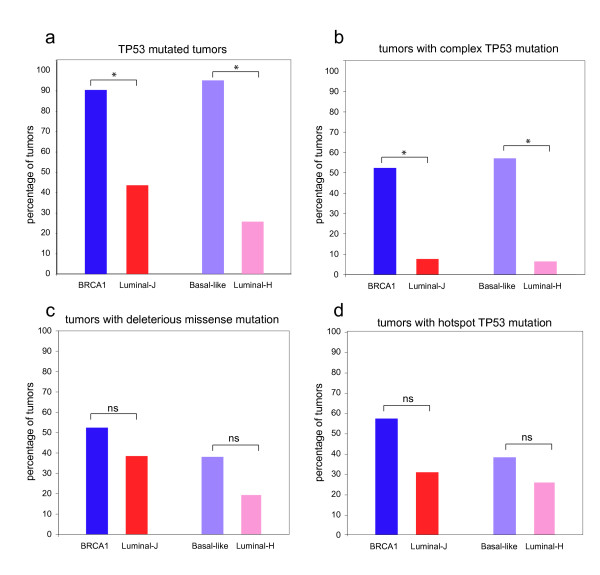
**Analysis of TP53 mutations in BRCA1-mutated tumors and BLBCs**. *TP53 *exons 2-9 were sequenced for 21/27 of the *BRCA1*-mutated tumors and for 13/21 of the luminal-J tumors. *TP53 *exons 2-11 were sequenced for all 21 BLBCs and 31 luminal-H tumors (see Table 1 for *TP53 *mutations see Additional File 1 for *TP53 *mutation frequencies). **a**. Amount of tumors with at least one *TP53 *mutation. **b**. Amount of tumors with at least one complex, predicted truncating *TP53 *mutation (frameshift, splice and nonsense mutations and in-frame insertions/deletions). **c**. Amount of tumors with at least one deleterious missense mutation. **d**. the amount of tumors with at least one hotspot mutation as defined by Walker *et al. *Three *BRCA1*-mutated tumors have a complex/truncating TP53 mutation and also a deleterious missense mutation. * Significant difference between groups (p < 0.01, determined with a Fisher's Exact Test), ns: no significant difference between groups.

Similar to the 90% (19/21) of *BRCA1*-mutated tumors, 95% (20/21) of the BLBCs harbored *TP53 *mutations, significantly more than the 46% (6/14) of the luminal-J and the 26% (8/31) of the luminal-H tumors (p = 7 × 10^-3 ^and p = 5 × 10^-7 ^respectively, two-tailed Fisher's Exact test), Figure 1 Additional File 2. We next compared *TP53 *mutation *types *from *BRCA1*-mutated tumors and non-hereditary BLBCs with luminal-H and luminal-J tumors. We found that 52% (11/21) of the *BRCA1*-mutated tumors and 57% (12/21) of the BLBCs have complex/truncating *TP53 *mutations, which is significantly more than the 8% (1/13) and 7% (2/31) of the luminal-J and luminal-H tumors, respectively (p = 1 × 10^-2 ^and p = 8 × 10^-5 ^respectively; two-tailed Fisher's Exact test, Figure 1 Additional File 2), indicating that this feature is common in hereditary *BRCA1*-mutated breast tumors and non-hereditary BLBCs. Interestingly, the increase in deleterious missense or hotspot *TP53 *mutations in *BRCA1*-mutated or BLBCs (8/21) their respective luminal tumor groups was not significant. Deleterious missense mutations: 11/21 *BRCA1*-mutated vs. 5/13 luminal-J, p = 5 × 10^-1^, and 8/21 BLBCs vs. 6/31 luminal-H tumors p = 2 × 10^-1^, two-tailed Fisher's Exact test). Hotspot mutations: 9/21 *BRCA1*-mutated vs. 3/13 luminal-J, p = 3 × 10^-1^, and 6/21 BLBCs vs. 6/31 luminal-H tumors p = 5 × 10^-1^, two-tailed Fisher's Exact test). Together, these data suggest that the increase of *TP53 *mutations in the *BRCA1*-mutated tumors and BLBCs is primarily due to the increase in complex/truncating *TP53 *mutations.

### Comparison of aCGH profiles of BRCA1-mutated tumors, BLBCs and luminal breast cancers

**Figure 2:**
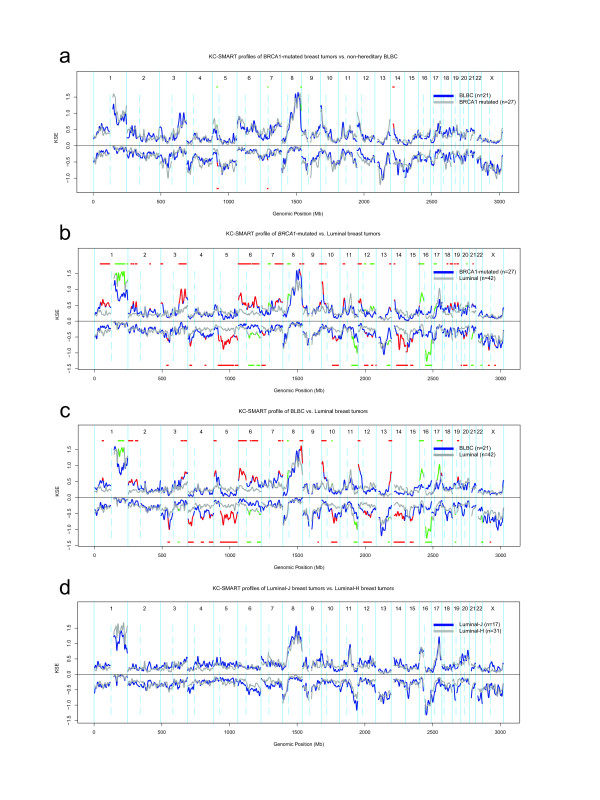
**Comparative-KC-SMART analysis of aCGH data from BRCA1-mutated tumors and BLBCs**. For each tumor group, *comparative*-KC-SMART was applied to normalized aCGH data, which was scaled to have a mean of zero and a standard deviation of one. The KSE curves for each tumor group are shown for gains and losses separately. **a**: *BRCA1*-mutated breast tumors (blue) vs. BLBCs (gray). CNAs that occur more often in the *BRCA1*-mutated tumors vs. the BLBCs are shown as red horizontal bars on above or below the KSE_gains _and KSE_losses _respectively, and they are plotted as red overlays on the blue KSE curves. CNAs that occur more often the BLBCs vs. the *BRCA1*-mutated tumors are shown as green horizontal bars above or below the KSE_gains _and KSE_losses _respectively, and as green overlays on the gray KSE curves. **b**: *BRCA1*-mutated tumors (blue) vs. luminal tumors (gray) c: BLBCs (blue) vs. luminal (gray) tumors. d: Luminal-J tumors (blue) vs. luminal-H tumors (gray).

To identify DNA copy number aberrations (CNAs) that occur significantly more often in *BRCA1*-mutated breast tumors than in BLBCs, we analyzed their aCGH with *comparative*-KC-SMART, a computational method for detection of genomic regions that have a significantly different aCGH signal *between two tumor groups*[35]. However, the DNA samples used to acquire aCGH profiles for the BLBC and the *BRCA1*-mutated tumor groups were isolated from fresh frozen tissue and FFPE material respectively, resulting in differences in log2-ratio distribution of the aCGH profiles (Additional File 1). To account for this difference, we normalized the data as explained in the Methods section. We applied *comparative*-KC-SMART to the normalized aCGH data of the *BRCA1*-mutated tumor group and the BLBC group (Figure 2a). *Comparative*-KC-SMART detected small CNAs on chromosomes 5, 7, 8 and 14 that are significantly different between these tumor groups (for regions and cancer-related genes see Additional File 3). BRCA1-specific losses and BLBC-specific gains on chromosomes 5 and 7 flanked each other and seem to be dependent on each other. Interestingly, the BRCA1-specific chromosome 7 loss encompasses *EGFR*. The BRCA1-specific gain on chromosome 14 peaks at the T-cell receptor alpha (*TCRα*) locus and encompasses, among other cancer-related genes, poly(ADP-ribose) polymerase 2 (*PARP2*), and B-cell lymphoma 2 like 2 (*BCL2L2*). Whether any of the genes located within these CNAs promote survival of BRCA1-deficient cells remains to be established.

Next, we used *comparative*-KC-SMART to compared CNAs in the *BRCA1*-mutated tumors and BLBCs vs. the combined luminal tumors (Figure 2b-c). Differential gains and losses are shown in Additional File 4. We compared CNAs between Luminal-J and luminal-H tumors as a control for merging these tumor groups (Figure 2d): as expected, no differences between the two luminal groups were detected.

Gains on chromosomes 1q and 16p, and the loss on chromosome 16q occur more often in the luminal tumors than in the basal-like/*BRCA1*-mutated tumors (Figure 2b-c, Table 2). The first chromosome 1q gain peaks at 177.31 Mb; the second 1q gain peaks at 202.65 Mb close to *MDM4 *(202.81 Mb). Similarly, the 16p gain peaks at 15.7 Mb. The chromosome 16q loss consists of two peaks: the first peak maps to 52.19 Mb, at the *BRD7*, *CYLD*, and *RBL2/p130 genes*. The second peak maps to 79.19 Mb, at one of the most active common fragile sites in the human genome, FRA16 D, associated with the *WWOX *gene (77.24 Mb). Many of the luminal tumors show a co-occurrence of chromosome 1q gain and 16q loss (Additional File 5).

**Table 2:** Overlapping gains and losses that differentiate BLBCs and BRCA1-mutated tumors from luminal breast tumors

a	Chr - region	Start (Mb)	End (Mb)	peaks (Mb) BRCA1-related	peaks (Mb) BLBC
Gains	1p	58.05	65.50	62.00	61.20
	2p-1	23.35	25.95	*26.45*	*27.45*
	2p-2	56.80	65.20	60.85	63.95
	3q-1	151.00	161.10	*150.85*	154.85
	3q-2	175.80	186.40	178.55	179.80
	6p-1	4.30	29.75	10.90, 19.20	14.05
	6p-2	37.05	58.65	42.60, 53.85	*36.65*
	6q-1	90.35	90.75	*86.50*	*91.00*
	6q-2	105.20	112.30	107.15	107.75
	6q-3	115.00	120.40	no peak in region	
	6q-4	123.55	138.75	125.55	135.60
	7q-1	132.80	139.40	134.40	*130.25*
	7q-2	155.15	157.65	156.95	157.65
	8q	127.40	132.95	*120.95*	*121.95, 135.3*
	10p-1	1.30	12.45	5.10	6.45
	10p-2	25.65	30.70	*24.90*	29.20
	12p	0.25	11.60	0.25, *16.10*	0.25
	13q	101.25	107.00	*99.55*	*110.35*
	19q	36.50	41.75	39.15	41.10

Losses	3p	53.00	53.25	*53.85*	*62.55*
	4p	15.65	27.05	18.55	*11.00*, 26.65
	5q-1	50.05	146.95	57.70, 70.75, 89.75,102.30, 116.25, 133.80	70.05, 89.85, 108.60,118.55, 136.30
	5q-2	161.40	171.20	*161.05, 178.30*	*157.30*
	10q-1	80.65	95.50	83.30, 90.15	91.05
	10q-2	105.55	111.35	109.70	108.60
	12q-1	47.70	48.35	no peak in region	*42.15*
	12q-2	54.30	59.25	55.25	58.40
	14q-1	38.30	44.75	40.65	*36.35*
	14q-2	48.35	92.95	57.35, 79.90, *98.00*	55.20, 66.15, 81.0, *95.05*
	15q	35.10	49.65	44.40	*33.70*, 42.85

**b**	**Chr - region**	**Start (Mb)**	**End (Mb)**	**peaks (Mb)**	

Gains	1q	176.70	215.40	177.35, 202.75	
	8p	35.95	38.90	41.5	
	16p	4.30	27.75	15.8	

Losses	6q-1	79.05	87.25	82.35	
	6q-2	142.75	149.25	no peak in region	
	6q-3	156.90	157.85	*160.05*	
	11q	104.45	125.35	112.30, *126.60*	
	13q	91.70	95.50	*99.1*	
	16q	45.15	88.50	52.30, 79.40	

Table 2: a. Overlapping differential CNAs of BLBCs and *BRCA1*-mutated breast tumors vs luminal breast tumors. b. Overlapping differential CNAs of luminal breast tumors vs BRCA1-mutated breast tumors and BLBCs.

Differential gains and losses determined by *comparative*-KC-SMART (Figure 1b-c). **a) **Overlapping regions that differentiate *BRCA1*-mutated tumors and BLBCs from luminal tumors. **b) **overlapping regions that differentiate luminal-J and luminal-H tumors from BLBC/*BRCA1*-mutated tumors. KSE peak locations are listed for all tumor groups; peaks in italics fall just outside the region of overlap.

### Impact of TP53 mutation on luminal tumors

**Figure 3:**
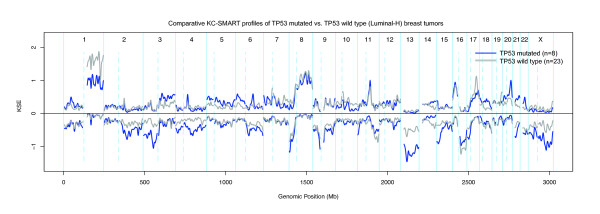
**Comparative-KC-SMART analysis of aCGH data from luminal breast tumors**. The luminal-H tumor group was divided into 8 *TP53*-mutated tumors and 23 *TP53 *wild-type tumors. KSE curves of the *TP53*-mutated tumors (blue) and the *TP53 *wild-type tumors (gray) are shown for gains and losses separately. *Comparative*-KC-SMART analysis did not detect significant differences between the two tumor groups.

Because BLBCs and *BRCA1*-mutated tumors are almost always *TP53*-mutated, we investigated whether *TP53 *mutations are associated with specific CNAs in luminal tumors. We stratified the 31 luminal-H tumors by their *TP53 *mutation status and used *comparative*-KC-SMART to compare both tumor groups. Although no differences were detected between CNAs from 8 *TP53*-mutated and 23 *TP53 *wild-type luminal breast tumors, quantitative differences in KC-SMART profiles can be observed, Figure 3. *TP53*-mutated tumors have more overall gains on chromosomes 3q, 6p, 20q, 21q and 22q and losses on chromosomes 2q, 3p, 4p, 4q, 13q, 15q and X. On the other hand, *TP53 *wild-type tumors have a higher incidence of chromosome 1q gain, and 16q loss. However, these data do not provide evidence for an altered profile of *TP53*-mutated luminal breast tumors, perhaps because the *TP53*-mutated tumor group is too small for robust statistical analysis.

### Analysis of genomic instability in BRCA1-mutated tumors and BLBCs

**Figure 4:**
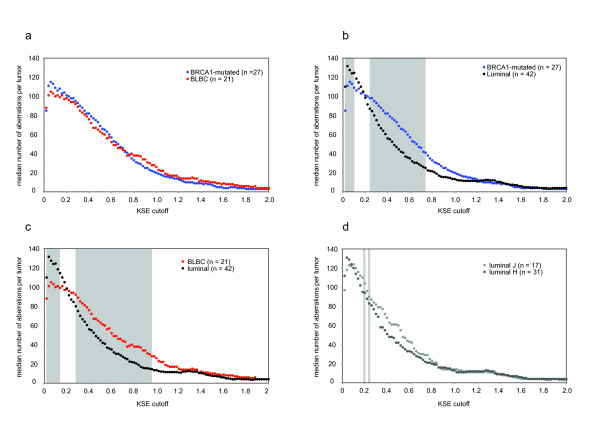
**Median number of aberrations**. **a**: Median amount of aberrations of *BRCA1*-mutated tumors and BLBCs. Normalized aCGH profiles from individual tumors were smoothed using KC-SMART (kernel width: 20 Mb) for a range of thresholds (KSE cutoff, x-axis). Gains exceeding a positive threshold and losses exceeding the same negative threshold were counted and the median was calculated over each tumor group. Gray background indicates thresholds for which the average number of CNAs in the BLBC group is significantly different compared with the luminal tumors, calculated with a two sided t-test (P < 0.01). Median amount of aberrations of **(b) ***BRCA1*-mutated tumors (blue dots) and luminal tumors (black dots); **(c) **BLBCs (red dots) and luminal breast tumors (black dots); **(d) **luminal-H (dark gray dots) and luminal-J tumors (light gray dots).

To determine the amount of CNAs in the different tumor groups, we used KC-SMART to smooth individual aCGH profiles and counted the amount of CNAs exceeding a range of cutoffs for each tumor separately. We found that the median amount of CNAs of BRCA1-mutated tumors was not different from the amount of CNAs found in BLBCs (Figure 4a). In contrast, we found that the median amount of CNAs is significantly greater in the *BRCA1*-mutated tumors compared with luminal breast tumors between KSE cutoffs 0.02 and 0.1 and between 0.24 and 0.74 (P < 0.01, two sided t-test Figure 4b). Similarly, the median amount of CNAs in the BLBCs was higher compared with luminal tumors for KSE cutoffs between 0.02 and 0.14 and between 0.28 and 0.96 (Figure 4c). We did not detect differences in the median amount of aberrations between luminal-J breast tumors and luminal-H except for KSE cutoffs 0.2 and 0.24 (Figure 4d).

### Overlapping gains and losses specific for BLBCs and BRCA1-mutated tumors

We compared gains and losses specific for the BLBC/*BRCA1*-mutated tumor groups relative to the luminal tumor groups. The BLBC/*BRCA1*-mutated tumors harbored overlapping differential gains on chromosomes 1p, 2p, 3q, 6p, 6q, 7q, 8q, 10p, 12p, 13q and 19q and losses on chromosomes 3p, 4p, 5q, 10q, 12q, 14q and 15q. The luminal tumor groups contained overlapping gains on chromosomes 1q, 8p and 16p, and an overlapping loss on chromosome 6q, 11q, 13q and 16q (Table 2).

Clearly, the differentially occurring CNAs detected by *comparative*-KC-SMART are fully dependent on the tumors included in the groups. However, when peaks of recurrent aberrations of two different analyses map closely together, this could point to a region whose gain or loss is relevant for tumorigenesis. Cancer-related genes that map to overlapping differential gains or losses between *BRCA1*-mutated tumors and BLBCs vs. luminal tumors are shown in Additional File 6.

### Unsupervised hierarchical clustering of breast cancers

**Figure 5:**
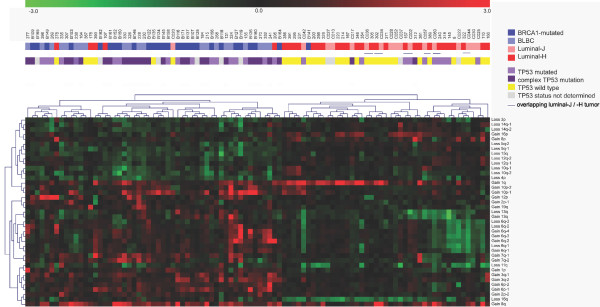
**Clustering analysis**. Unsupervised hierarchical clustering of 27 BRCA1-mutated tumors, 21 BLBCs, 17 luminal-J tumors and 31 luminal-H tumors. For each individual tumor, a KSE curve was calculated by smoothing each tumor profile with KC-SMART. A mean KSE value for was determined for all overlapping regions of gain and loss (shown in Table 2) between BLBCs (light blue) and BRCA1-mutated tumors (dark blue), and for the overlapping regions between the luminal-H (red) and luminal-J (pink) groups. We used two-dimensional Pearson correlation to perform complete linkage clustering over the mean KSE values and tumors.

The overlapping differential gains and losses of BLBCs and *BRCA1*-mutated tumors may represent regions that discriminate *BRCA1*-mutated tumors and BLBCs from luminal tumors. To test this possibility, we performed an unsupervised hierarchical clustering analysis. First, we smoothed each tumor profile with KC-SMART to remove experimental noise. Then, for each of the regional aberrations specific for the BLBC/*BRCA1*-mutated tumors or the luminal tumors (shown in Table 2), we calculated the mean of all KSE values within the region for all tumors. We used the mean KSE values to perform a hierarchical clustering of samples and regions using complete linkage and pearson correlation (Figure 5). The tumors clustered in two branches: 6 luminal tumors and 47 of the 48 BLBCs/*BRCA1*-mutated tumors clustered in one branch, whereas 1 *BRCA1*-mutated tumor and 42 of the 48 luminal-H/J tumors clustered in the other branch. Interestingly, the BRCA1 and BLBC cases do not form separate clusters but mix together, meaning that a limited amount of regions can distinguish BLBCs and *BRCA1*-mutated tumors from luminal tumors. The fact that the luminal-H and luminal-J tumors are mixed, shows that no unwanted biases are introduced by differences in quality of DNA from FFPE vs. fresh-frozen tumor material. As an additional internal control we have used the 6 luminal tumors for which DNA from FFPE and fresh-frozen tumor tissue was both available, and each of the 6 pairs cluster together. Notably, four of the six *TP53 *wild-type tumors that clustered within the BLBC/*BRCA1*-mutated branch were luminal. One of the six luminal tumors that clustered within the BLBC/*BRCA1*-mutated branch also had a complex *TP53 *mutation, whereas only two of the luminal tumors clustering in the luminal branch had a complex *TP53 *mutation.

## Discussion

BLBC is an aggressive subgroup of breast cancers for which, so far, no druggable target has been identified. In recent years it has become clear that phenotypic features of a subset of BLBCs resemble those of hereditary *BRCA1*-mutated breast cancers [13] and are distinctly different from the more common luminal breast tumors. *BRCA1*-mutated breast tumors are HR deficient (HRD) and can therefore be targeted with DNA-damaging agents or PARP inhibitors [38,39]. Because there is evidence that a substantial fraction of BLBCs have HR pathway defects [40], we set out to determine which molecular characteristics of *BRCA1*-mutated tumors are common to BLBCs but not to luminal tumors. Since some of these characteristics are likely to be linked to BRCA1-disfunction or the HRD phenotype, they might provide important leads for discovery of new biomarkers or drug targets.

### High incidence of protein truncating TP53 mutations in BLBC and BRCA1-mutated breast cancer

We and others have previously shown that *BRCA1*-mutated breast tumors exhibit an increased frequency of *TP53 *mutations due to a selective increase in complex *TP53 *mutations such as frameshift, nonsense and splice mutations or in-frame insertions/deletions [10,23]. In this study, we found that almost all *BRCA1*-mutated tumors and BLBCs are *TP53 *mutated compared with 25-50% of the luminal-tumors. Interestingly, 52.4% of the *BRCA1*-mutated breast tumors and 57.1% of the BLBCs have complex *TP53 *mutations, significantly more compared with ~7% luminal tumors. It has been suggested that the increased incidence of complex *TP53 *mutations in *BRCA1*-mutated tumors is a direct consequence of the genomic instability resulting from the DNA repair defect induced by BRCA1 loss [10,23]. Furthermore, the DSB repair defect of BRCA1-deficient tumors might confer strong selection pressure on mutation of *TP53 *in order to abrogate the p53-dependent DNA damage checkpoint [41]. The high frequency of *TP53 *mutations in non-hereditary BLBCs might suggest that these tumors are also compromised in homology-directed DSB repair. To test this possibility, it would be interesting to perform functional assays to measure DNA damage response and DNA repair in non-hereditary BLBCs with a (complex) TP53 mutation. It has previously been suggested that *TP53 *mutations, including complex *TP53 *mutations, affecting the DNA binding domain of the p53 protein may cause resistance to several different cytotoxic compounds such as anthracyclins, 5FU and mitomycin [42,43]. However, tumors used in these studies were primarily invasive ductal carcinomas, most of which are likely not compromised in homology directed DSB repair. It is therefore interesting that Silver *et al*. recently reported a significant association between truncating *TP53 *mutations and cisplatin response in TNBCs [44].

It is interesting that, like us, Manie *et al.*, found an increased frequency of complex *TP53 *mutations in *BRCA1*-mutated breast tumors; however, they did not detect this feature in BLBCs [10,23]. It is possible that BLBCs are more effectively identified by gene expression profiling than by immunohistochemical selection of tumors negative for ER/PR/HER2 and positive for CK5/6, CK14 or EGFR, as done by Manie *et al*. Of note, the *TP53 *mutation analyses for the *BRCA1*-mutated breast tumors and the BLBCs described in this manuscript were performed in different labs, thereby reducing the possibility that the detection of a high incidence of complex/truncating *TP53 *mutations is due to a methodical artifact.

### Recurrent CNAs in luminal tumors

Luminal breast tumors are mostly *TP53 *wild type, or they have a common *TP53 *hotspot mutation. Interestingly, the chromosome 1q gain and 16q loss (1q/16q) differentiate luminal tumors from *BRCA1*-mutated/BLBC tumors. Co-occurrence of these aberrations results from an unbalanced translocation event [45,46] and has been associated with *TP53 *wild-type status, low tumor grade and good prognosis [47]. Indeed, many of the ER-positive luminal tumors show the 1q/16q co-occurrence. The peak of the chromosome 1q gain of the luminal tumor group maps to 202.75 Mb, with the *MDM4 *gene at (202.81 Mb), which is a negative regulator of p53 [48]. The 16q loss has two peaks, the first maps near the bromodomain 7 (*BRD7*) gene, associated with downregulation of p53 [49], *CYLD*, loss of which is associated with oncogenesis by activation of NF-ΚB signaling [50], and the retinoblastoma-like 2 gene (*RBL2/p130*), involved in G_1_S cell cycle control and senescence [51]. The second peak on the 16q loss maps to one of the most active common fragile sites in the human genome, FRA16 D (associated with the *WWOX *gene), which could underlie the translocation process [52]. Together, these data suggest that, whereas development of BLBCs or *BRCA1*-mutated tumors depends on *TP53 *mutation, indirect p53 downregulation may be sufficient for luminal tumor development.

### aCGH profiles of BRCA1-mutated breast tumors resemble BLBCs

*BRCA1*-mutated breast tumors are associated with a specific aCGH profile which exhibits features that can be used to identify hereditary breast tumors for which information on *BRCA1*-mutation is not available [19,22,24]. Several previous studies have reported a specific aCGH profile for BLBCs different from other breast cancer subtypes [53,54]. Interestingly, comparison of CNAs from *BRCA1*-mutated tumors and BLBCs using *comparative*-KC-SMART yielded a limited set BRCA1- or BLBC-specific aberrations. The peaks of most gains and losses of *BRCA1*-mutated tumors and BLBCs co-localized, suggesting a common selection pressure during development of these tumors. Indeed, we found that *BRCA1*-mutated tumors and BLBCs showed many overlapping CNAs, including the chromosome 3q gain and the chromosome 5q loss. Importantly, clustering on the basis of these regions separated BLBCs and *BRCA1*-mutated breast tumors from luminal breast tumors.

### BRCA1 and TP53 involvement in the BLBC phenotype

Our data show that *BRCA1*-mutated tumors share molecular characteristics of undifferentiated BLBCs. It has previously been proposed that *BRCA1*-mutation is associated with BLBC because BRCA1 function has stem cell regulation properties and because loss of BRCA1 impairs DNA damage repair during epithelial cell differentiation [55]. However, it is also possible that a defect in DNA repair mechanisms is primarily harmful in proliferating cells, which are more prone to acquire genetic lesions during cell division. Notably, proliferating cells in the premenopausal mammary gland have been shown to rarely express hormone receptors whereas hormone receptor-positive cells only rarely divide [56]. In line with this, and in contrast to most breast tumors, both *BRCA1*-mutated tumors and BLBCs often occur in premenopausal breast epithelia [57]. Therefore, we propose that inadequate DNA repair mechanisms result in increased susceptibility to genomic instability in the proliferating hormone receptor-negative cells of the premenopausal mammary gland. Furthermore, we propose that because of this, hormone receptor-negative cells depend heavily on p53-mediated cell cycle arrest and apoptosis to remain untransformed. This line of thought lends great importance to *TP53 *mutation in TNBC.

## Conclusions

Our data suggest that a small subset of genomic regions may be useful for the identification of BRCA1-like BLBCs, which exhibit a high frequency of *TP53 *mutations, especially protein truncating mutations. These features of basal-like breast cancers might be useful for the identification of tumors with increased sensitivity to (high-dose or dose-dense) alkylating agents and PARP inhibitors. In support of this, it was recently reported that *TP53 *mutations in non-inflammatory BLBCs are highly predictive of complete response to dose-dense neoadjuvant chemotherapy with epirubicine-cyclophosphamide [58]. Furthermore, a significant positive correlation was found between truncating *TP53 *mutations and cisplatin response in TNBCs [44]. Together, these and our data support further investigation of (protein truncating) *TP53 *mutation status as a potential predictor of chemotherapy responsiveness in solid tumors.

## Competing interests

The authors declare that they have no competing interests.

## Authors' contributions

HH (first author) designed, performed the analyses and wrote the manuscript, HH (second author) and MJV provided tumor information of the Horlings tumor panel [25]. AV provided help with statistical analyses and performed the bioinformatics. AL and ALB-D sequenced TP53 from the tumor panel described by Horlings et al [25] and helped with TP53 mutation interpretation, PN provided tumor information from the Joosse tumor panel [24]. JJ supervised the project. All authors read and approved the manuscript.

## Pre-publication history

The pre-publication history for this paper can be accessed here:

http://www.biomedcentral.com/1471-2407/10/654/prepub

## Supplementary Material

Additional file 1 **fresh frozen tissue vs. FFPE**. When comparing the aCGH profiles acquired by hybridization of DNA isolated from formalin fixed paraffin embedded tissue (FFPE) and fresh-frozen tissues we noticed that the log2 ratios obtained from the different DNA sources have different distributions. Therefore, we transformed both the FFPE and the fresh frozen datasets to have a mean of zero and a standard deviation of one, using all tumors (both luminal and basal-like) from the Horlings dataset, and all tumors (both *BRCA1*-mutated and luminal) from the Joosse dataset. The influence of this transformation is shown for the six tumors that were included in the aCGH datasets from both the luminal-H, and luminal-J tumor groups, hybridized from DNA isolated from fresh frozen tissue and FFPE material respectively. For each tumor, we compared the log2 ratios from both platforms. Red line: x = y (if log2 ratios of FFPE and fresh frozen tumor data would be equal), Blue line: ratio of the factors used to scale both datasets to a standard deviation of 1. Click here for file

Additional file 2 **Frequency of TP53 mutations in BRCA1-mutated tumors and BLBCs**. *TP53 *exons 2-9 were sequenced for 21/27 of the *BRCA1*-mutated tumors and for 13/21 of the luminal-J tumors. *TP53 *exons 2-11 were sequenced for all BLBCs and luminal-H tumors (for *TP53 *mutation data, see Table 1). Click here for file

Additional file 3 **Regions of differential gains and losses detected by *comparative*-KC-SMART analyses between the BRCA1-mutated tumors and BLBCs**. Genes that map within these regions and locations of the KSE peaks. Click here for file

Additional file 4 **Regions of differential gains and losses detected by *comparative*-KC-SMART analyses in (a) the BRCA1-mutated tumors and BLBCs vs. luminal tumors, genes in overlapping regions are shown in green**. KSE peak locations are given for both tumor groups (**b**) luminal tumors vs. *BRCA1*-mutated and BLBC tumor groups, KSE peak locations are given for both tumor groups. Click here for file

Additional file 5 **Line plots**. Normalized aCGH profiles of each individual tumor were smoothed with KC-SMART. Normalization was done by transformation of the log2 ratios from the FFPE aCGH dataset (i.e. all log2 ratios from BRCA1-mutated and luminal-J tumors taken together) and the fresh frozen dataset (see Methods section). The position of gains and exceeding the standard deviation of 1 are shown in red, the position of losses exceeding the standard deviation of -1 are shown in blue. Click here for file

Additional file 6 **Cancer related genes in overlapping gains and losses found by comparative-KC-SMART**. Cancer-related genes that map to the differential gains and losses determined by *comparative*-KC-SMART (Figure 2b/c). **a) **Cancer related genes that map in the overlapping regions that differentiate *BRCA1*-mutated and BLBC tumors from luminal tumors. **b) **Cancer related genes that map in the overlapping regions that differentiate luminal tumors from BLBC/BRCA1-mutated tumors. KSE peak locations are listed for all tumor groups, peaks in italics fall just outside the region of overlap. Cancer related genes are taken from the Atlas of Genetics and Cytogenetics in Oncology and Haematology [59] and the cancer gene census [60]. The cancer related genes closest to the KSE peak locations are shown in red for the *BRCA1*-mutated tumors, and in blue for the BLBCs tumors. When the same gene maps closest the peaks of both KSE-curves it is shown in green. For the luminal tumor group, cancer related genes closest to the KSE peak locations are shown in bold. Click here for file
