# Supplementary figures and images for: BRCA1-mutated and basal-like breast cancers have similar aCGH profiles and a high incidence of protein truncating TP53 mutations
Source: BMC Cancer. 2010 Nov 30;10:654. doi: 10.1186/1471-2407-10-654 (PMC3002929; doi:10.1186/1471-2407-10-654)

C036

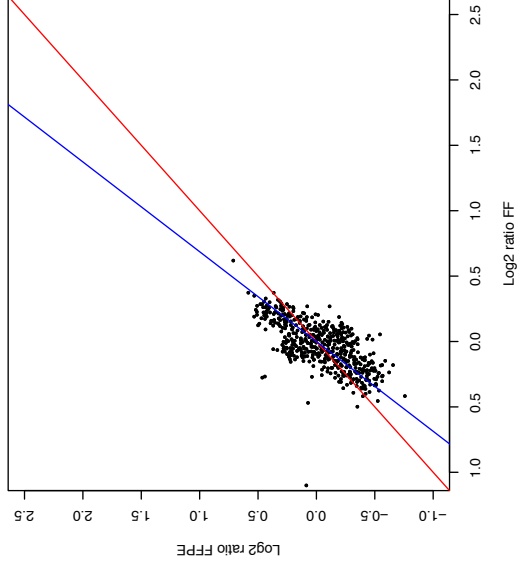

C060

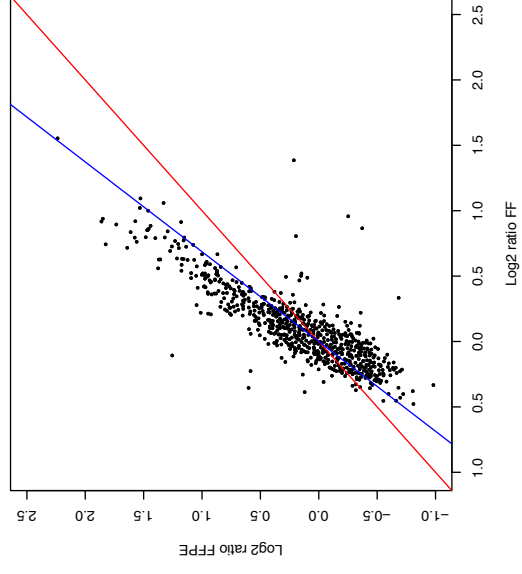

C034

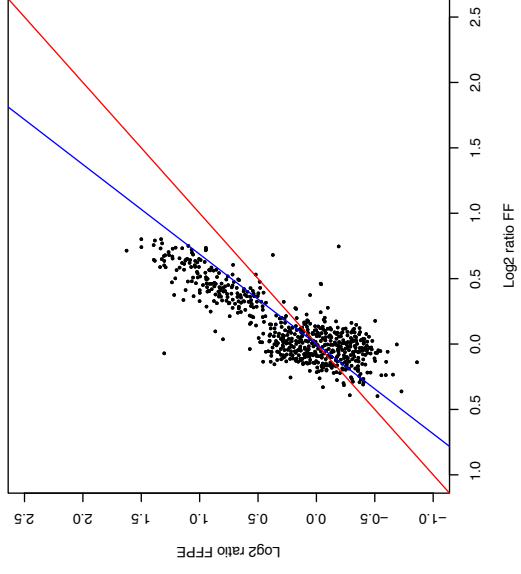

C057

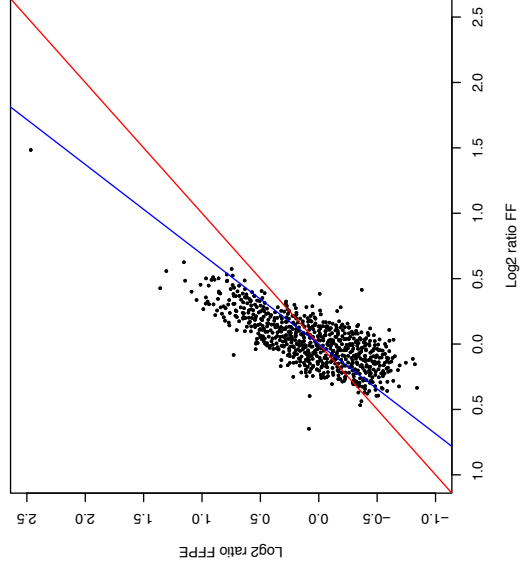

C002

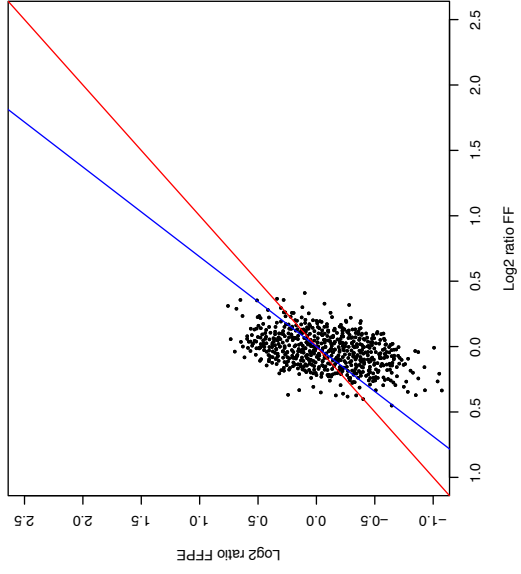

C044

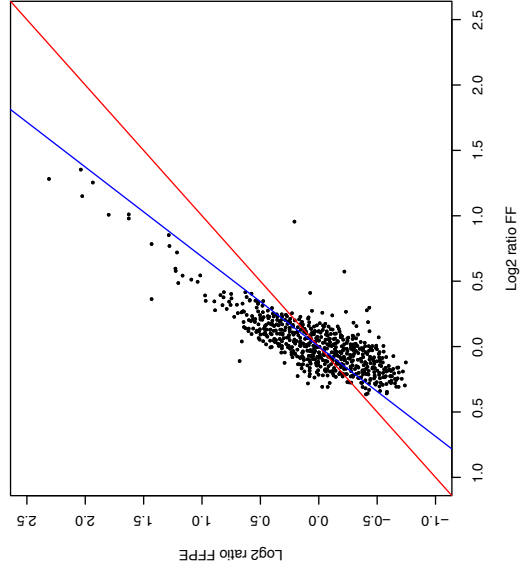

Supplement: Additional file 1 — fresh frozen tissue vs. FFPE. When comparing the aCGH profiles acquired by hybridization of DNA isolated from formalin fixed paraffin embedded tissue (FFPE) and fresh-frozen tissues we noticed that the log2 ratios obtained from the different DNA sources have different distributions. Therefore, we transformed both the FFPE and the fresh frozen datasets to have a mean of zero and a standard deviation of one, using all tumors (both luminal and basal-like) from the Horlings dataset, and all tumors (both BRCA1-mutated and luminal) from the Joosse dataset. The influence of this transformation is shown for the six tumors that were included in the aCGH datasets from both the luminal-H, and luminal-J tumor groups, hybridized from DNA isolated from fresh frozen tissue and FFPE material respectively. For each tumor, we compared the log2 ratios from both platforms. Red line: x = y (if log2 ratios of FFPE and fresh frozen tumor data would be equal), Blue line: ratio of the factors used to scale both datasets to a standard deviation of 1. [file 1471-2407-10-654-S1.PDF]

BRCA1-mutated breast tumors (n=27)

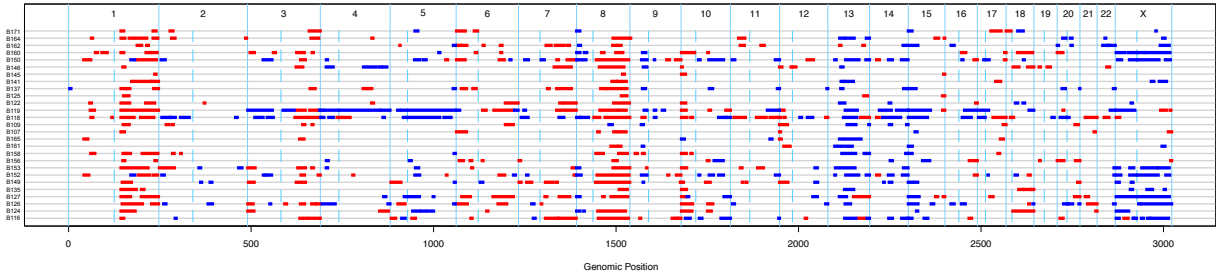

BLBCs (n=21)

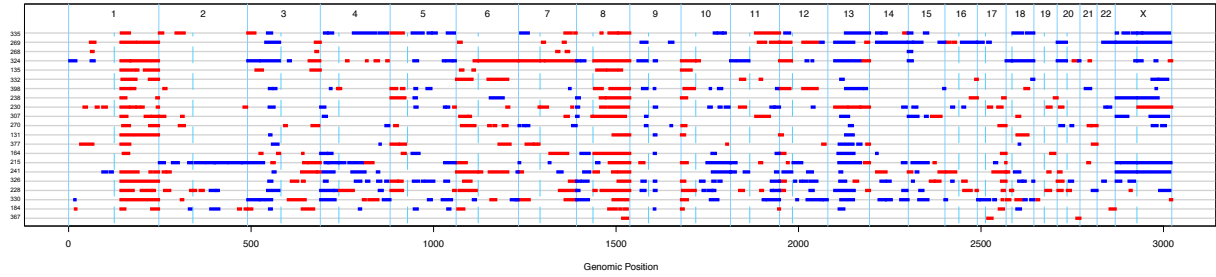

Luminal-J breast tumors (n=17)

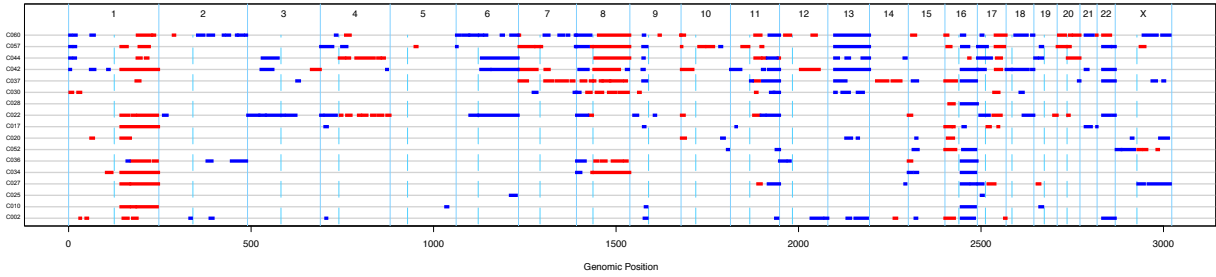

Luminal-H breast tumors (n=31)

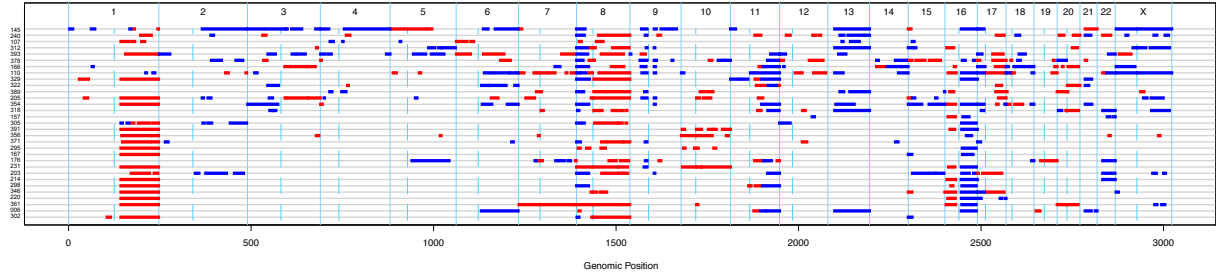

Supplement: Additional file 5 — Line plots. Normalized aCGH profiles of each individual tumor were smoothed with KC-SMART. Normalization was done by transformation of the log2 ratios from the FFPE aCGH dataset (i.e. all log2 ratios from BRCA1-mutated and luminal-J tumors taken together) and the fresh frozen dataset (see Methods section). The position of gains and exceeding the standard deviation of 1 are shown in red, the position of losses exceeding the standard deviation of -1 are shown in blue. [file 1471-2407-10-654-S5.PDF]
