# Supplementary material for: BRCA1-mutated and basal-like breast cancers have similar aCGH profiles and a high incidence of protein truncating TP53 mutations
Source: BMC Cancer. 2010 Nov 30;10:654. doi: 10.1186/1471-2407-10-654 (PMC3002929; doi:10.1186/1471-2407-10-654)
Supplement: Additional file 2 — Frequency of TP53 mutations in BRCA1-mutated tumors and BLBCs. TP53 exons 2-9 were sequenced for 21/27 of the BRCA1-mutated tumors and for 13/21 of the luminal-J tumors. TP53 exons 2-11 were sequenced for all BLBCs and luminal-H tumors (for TP53 mutation data, see Table 1). [file 1471-2407-10-654-S2.PDF]

Additional File 2 *TP53* mutations in BRCA1-mutated and BLBC compared with luminal breast tumors

Tumors group	total	*TP53* mutated tumors			tumors with a complex *TP53* mutation[1]			tumors with a deleterious missense *TP53* mutation[2]			tumors with a hotspot *TP53* mutation[3]		
	n	n	%	p[4]	n	%	p	n	%	p	n	%	p
BRCA1-mutated	21	19	90.50%	7.40E-03	11	52.4%	1.07E-02	11	52.40%	4.96E-01	9	42.90%	2.92E-01
Luminal-J	13	6	46.20%		1	7.7%		5	38.50%		3	23.10%	
BLBC	21	20	95.20%	4.80E-07	12	57.1%	8.09E-05	8	38.10%	2.03E-01	6	28.60%	5.12E-01
Luminal-H	31	8	25.80%		2	6.5%		6	19.30%		6	19.30%	

[1]Amount of tumors with at least one complex *TP53* mutation (truncating frameshift, splice and nonsense mutations and in-frame insertions/deletions).

[2]Amount of tumors with at least one deleterious missense mutation.

[3]Amount of tumors with at least one hotspot mutation.Three *BRCA1*-mutated tumors have a complex and a deleterious missense mutation.

[4]p values calculated with a two tailed Fisher's Exact test (http://faculty.vassar.edu/lowry/fisher.html)
