# Supplementary material for: BRCA1-mutated and basal-like breast cancers have similar aCGH profiles and a high incidence of protein truncating TP53 mutations
Source: BMC Cancer. 2010 Nov 30;10:654. doi: 10.1186/1471-2407-10-654 (PMC3002929; doi:10.1186/1471-2407-10-654)
Supplement: Additional file 3 — Regions of differential gains and losses detected by comparative-KC-SMART analyses between the BRCA1-mutated tumors and BLBCs. Genes that map within these regions and locations of the KSE peaks. [file 1471-2407-10-654-S3.PDF]

Cancer related genes in regions detected by *comparative*-KC-SMART

chromosome	start (Mb)	end (Mb)	cancer related genes in region	KSE peak
**BRCA1 gains**				
14	19.65	26.50	TTC5, CCNB1IP1, PARP2, TEP1, NP, APEX1, ANG, RNASE1, RNASE2, NDRG2, RNASE13, AL161668.6, SUPT16H, RAB2B, SALL2, **TCRα**, DAD1, OXA1L, MMP14, REM2, PRMT5, ACIN1, CEBPE, BCL2L2, PABPN1, EFS, CMTM5, MYH6, NGDN, DHRS4, LRRC16B, PSME1, PSME2, RNF31, TSSK4, NEDD8, GMPR2, TINF2, TGM1, CIDEB, LTB4R2, LTB4R, RIPK3, NFATC4, CMA1, CTSG, GZMB, STXBP6, NOVA1	21.95
**BRCA1 losses**				
5	35.25	41.55	PRLR, SKP2, SLC1A3, NIPBL, GDNF, LIFR, OSMR, AC026713.5, DAB2, **PTGER4,** PRKAA1, CARD6, C7, PLCXD3	40.60
7	54.50	56.55	SEC61G, EGFR, GBAS, CCT6A, **PHKG1**	56.25
**BLBC gains**				
5	33.10	35.20	ADAMTS12, SLC45A2, AMACR, RAD1	no peak
7	56.60	56.70	no cancer related genes in region	56.70
8	141.70	145.65	PTK2, PTP4A3, ARC, PSCA, LY6K, C8orf55, SLURP1, LYNX1, GML, LY6E, GLI4, MAFA, C8orf73, EEF1D, ZNF707, MAPK15, SCRIB, NRBP2, PLEC1, GRINA, GPAA1, CYC1, C8orf30A, BOP1, HSF1, SCRT1, ADCK5, SLC39A4, VPS28, GPT	no peak
