# Supplementary material for: BRCA1-mutated and basal-like breast cancers have similar aCGH profiles and a high incidence of protein truncating TP53 mutations
Source: BMC Cancer. 2010 Nov 30;10:654. doi: 10.1186/1471-2407-10-654 (PMC3002929; doi:10.1186/1471-2407-10-654)
Supplement: Additional file 4 — Regions of differential gains and losses detected by comparative-KC-SMART analyses in (a) the BRCA1-mutated tumors and BLBCs vs. luminal tumors, genes in overlapping regions are shown in green. KSE peak locations are given for both tumor groups (b) luminal tumors vs. BRCA1-mutated and BLBC tumor groups, KSE peak locations are given for both tumor groups. [file 1471-2407-10-654-S4.PDF]

a: Differential CNAs of BLBC and BRCA1-mutated breast tumors relative to luminal breast tumors

	BRCA1-mutated			BLBC		
**Chr.**	**Start (Mb)**	**End (Mb)**	**peaks (Mb)**	**Start (Mb)**	**End (Mb)**	**peaks (Mb)**
			**Gains**			
**1**	44.60<br>86.80	79.70<br>111.05	46.0, 62.0, 73.55<br>102.75	58.05	65.50	61.2
**2**	23.35<br>37.15<br>56.15<br>162.30	25.95<br>41.60<br>65.20<br>165.40	*26.45*<br>39.85<br>60.85<br>163.35	5.75<br>17.20<br>56.80	10.30<br>34.65<br>71.95	*11.20*<br>27.45<br>63.95
**3**	0.9<br>134.95<br>168.95	12.35<br>161.10<br>186.40	6.20<br>150.85<br>178.55	151.00<br>175.80	163.55<br>188.85	154.85<br>179.80, 188.80
**5**				2.65	16.80	13.85
**6**	1.30<br>37.05<br>67.75<br>87.30<br>104.50<br>115.00<br>149.30	29.75<br>58.65<br>78.10<br>90.75<br>112.30<br>138,75<br>151.95	10.90, 19.20<br>42.60, 53.85<br>74.95<br>*86.50*<br>107.15<br>125.55<br>150.90	4.30<br>90.35<br>105.20<br>123.55	58.65<br>93.70<br>120.40<br>142.70	14.05, 36.65<br>91.0<br>107.75<br>135.60
**7**	102.25<br>132.80<br>151.20	123.00<br>141.30<br>157.65	108.20, 116.65,<br>134.40<br>156.95	127.35<br>155.15	139.40<br>157.65	130.25<br>157.65
**8**	124.70	132.95	120.95	127.40	145.65	*121.95*, 135.3
**9**	1.05<br>125.00	7.55<br>133.75	1.05<br>128.80			
**10**	1.30<br>25.65	12.45<br>31.20	5.10<br>28.10	1.30<br>24.90	15.20<br>30.70	6.45<br>29.20
**11**	24.00	34.35	31.45			
**12**	0.25	21.80	0.25, 16.10	0.25	11.60	0.25
**13**	101.25	107.00	*99.55*	95.55	113.85	110.35
**14**	19.65	23.30	21.95			
**15**	98.30	99.60	99.6			
**17**				71.85	75.90	71.3
**18**	36.70<br>59.20<br>70.60	39.35<br>68.95<br>75.60	*40.30*<br>63.05<br>no peak			
**19**	15.85<br>33.00	18.90<br>41.75	16.80<br>39.15	36.50	44.10	41.1
**21**	24.60	25.50	20.55			
			**Losses**			
**3**	44.30	53.25	53.85	53.00	63.55	62.55
**4**	15.65<br>127.95	27.05<br>131.85	18.55<br>131.60	4.00<br>99.85<br>159.20<br>173.30	39.20<br>115.05<br>166.20<br>183.25	11.00, 26.65<br>*97.20*, 109.20,<br>163.80<br>181.05
**5**	33.55<br>50.05<br>161.40	42.85<br>146.95<br>178.30	40.60<br>57.70, 70.75, 89.75,<br>102.30, 116.25, 133.80<br>*161.05*, 178.30	50.05	171.20	70.05, 89.85, 108.60,<br>118.55, 136.30,<br>157.30
**7**	4.75	27.55	18.95			
**9**				114.10	115.55	120
**10**	80.65<br>105.50	95.50<br>122.35	83.30, 90.15<br>109.70	74.05<br>76.25<br>105.55	75.15<br>96.50<br>111.35	no peak<br>91.05<br>108.60
**12**	47.70<br>54.30<br>67.05<br>99.60<br>131.65	48.35<br>59.25<br>71.10<br>107.35<br>131.90	no peak<br>55.25<br>69.65<br>104.65<br>131.90	41.45<br>74.15<br>87.50	64.85<br>77.65<br>95.60	42.15, 58.40<br>75.95<br>90.15
**14**	38.30<br>48.35	44.75<br>105.35	40.65<br>57.35, 79.90, 98.00	19.65	92.95	19.65, 36.35, 55.20,<br>66.15, 81.0, *95.05*
**15**	0.20<br>35.10	10.9<br>49.65	*11.75*<br>44.40	31.45	51.35	33.70, 42.85
**20**	0.40<br>18.85<br>34.10	1.40<br>23.60<br>41.05	0.4<br>20.55<br>38.75			
**X**	42.35<br>89.45	46.80<br>92.80	46.10<br>*101.15*	55.55	57.60	55.1

b: Differential CNAs of luminal breast tumors relative to BRCA1-mutated breast cancers and BLBCs

	Luminal (vs. BRCA1-mutated)			Luminal (vs. BLBC)		
**Chr.**	**Start (Mb)**	**End (Mb)**	**peaks (Mb)**	**Start (Mb)**	**End (Mb)**	**peaks (Mb)**
			**Gains**			
**1**	154.55<br>171.35<br>242.30	163.55<br>224.15<br>246.05	161.15<br>177.35, 202.75, 217.80<br>246.05	176.7	215.4	177.35, 202.75,<br>*217.80*
**7**	52.60<br>62.40	56.70<br>67.00	56.7<br>62.40			
**8**	35.95<br>47.90	43.35<br>53.05	41.5<br>47.90	34.55	38.90	41.5
**10**				75.20	76.20	78.05
**12**	48.40<br>91.25<br>107.40	54.25<br>99.55<br>116.1	49.90<br>91.85<br>112.45			
**16**	4.30	31.05	15.8	0.15	27.75	15.8
**17**	21.10	33.55	26.10, *36.45*	42.65	62.50	57.45
**20**	41.10	42.95	no peak in region			
			**Losses**			
**3**				129.60	134.00	128.55
**6**	78.15<br>90.80<br>112.35<br>138.8<br>152.00	87.25<br>104.45<br>114.95<br>149.25<br>157.85	82.35<br>95.65<br>*117.25*<br><br>160.05	67.35<br>79.05<br>142.75<br>156.90	70.10<br>90.30<br>149.60<br>163.45	*70.30*<br>82.35<br>no peak<br>160.05
**11**	87.55	128.00	90.65, 112.30, 126.60	104.45	125.35	112.30, 126.60
**13**	82.06	101.20	84.85, 99.10	91.70	95.50	*84.85, 99.10*
**16**	45.15	88.50	52.30, 79.40	45.15	88.50	52.30, 79.40
**19**				20.45	23.60	21.05
**21**	14.70	24.55	14.7			
**22**	37.65	41.30	no peak in region	42.30	49.4	49.4
