# Supplementary material for: BRCA1-mutated and basal-like breast cancers have similar aCGH profiles and a high incidence of protein truncating TP53 mutations
Source: BMC Cancer. 2010 Nov 30;10:654. doi: 10.1186/1471-2407-10-654 (PMC3002929; doi:10.1186/1471-2407-10-654)
Supplement: Additional file 6 — Cancer related genes in overlapping gains and losses found by comparative-KC-SMART. Cancer-related genes that map to the differential gains and losses determined by comparative-KC-SMART (Figure 2b/c). a) Cancer related genes that map in the overlapping regions that differentiate BRCA1-mutated and BLBC tumors from luminal tumors. b) Cancer related genes that map in the overlapping regions that differentiate luminal tumors from BLBC/BRCA1-mutated tumors. KSE peak locations are listed for all tumor groups, peaks in italics fall just outside the region of overlap. Cancer related genes are taken from the Atlas of Genetics and Cytogenetics in Oncology and Haematology [59] and the cancer gene census [60]. The cancer related genes closest to the KSE peak locations are shown in red for the BRCA1-mutated tumors, and in blue for the BLBCs tumors. When the same gene maps closest the peaks of both KSE-curves it is shown in green. For the luminal tumor group, cancer related genes closest to the KSE peak locations are shown in bold. [file 1471-2407-10-654-S6.PDF]

Table 2a. Overlapping differential CNAs of BLBCs and BRCA1-mutated breast tumors vs. luminal breast tumors

Chr - region	Start (Mb)	End (Mb)	Cancer related genes in regions of overlap<br>peak locations of KSE curves:<br>red: KSE peaks of *BRCA1*-mutated group; blue: KSE peaks of BLBC group;<br>green: KSE peaks in both tumor groups co-occur	peaks (Mb)<br>BRCA1-related	peaks (Mb)<br>BLBC
**Gains**					
1p	58.05	65.50	DAB1, TACSTD2, JUN, **CYP2J2**, **USP1**, ANGPTL3, ATG4C, FOXD3, ITGB3BP, PGM1, ROR1, JAK1	62.00	61.20
2p-1	23.35	25.95	TP53I3, ITSN2, NCOA1, DNAJC27, POMC, DNMT3A	*26.45*	*27.45*
2p-2	56.80	65.20	VRK2, FANCL, **BCL11A**, REL, AHSA2, XPO1, B3GNT1, MDH1, **UGP2, VPS54**, PELI1	60.85	63.95
3q-1	151.00	161.10	RNF13, SIAH2, GPR87, AADAC, MBNL1, P2RY1, **RAP2B**, AC018452.11, DHX36, MME, GMPS, SSR3, CCNL1, PTX3, MLF1, RARRES1, IQCJ, IL12A	*150.85*	154.85
3q-2	175.80	186.40	**ZMAT3**, PIK3CA, GNB4, FXR1, DNAJC19, SOX2, DCUN1D1, LAMP3, MCF2L2, ABCC5, AP2M1, PSMD2, EIF4G1, THPO, CHRD, EPHB3, MAGEF1	178.55	179.80
6p-1	4.30	29.75	NRN1, F13A1, SSR1, RIOK1, DSP, BMP6, MUTED, EEF1E1, TFAP2A, AL358777.12, **MAK**, NEDD9, TMEM170B, EDN1, AL355137.23, RANBP9, **CD83**, JARID2, CAP2, NUP153, NHLRC1, TPMT, DEK, IBRDC2, **ID4,** E2F3, AL513015.6, SOX4, PRL, NRSN1, DCDC2, MRS2L, GPLD1, TTRAP, GMNN, SCGN, HIST1H1A, HFE, HIST1H4A, HIST1H2AC, BTN1A1, HMGN4, ABT1, HIST1H4I, ZKSCAN4, GPX5, TRIM27, MAS1L UBD, GABBR1	10.90, 19.20	14.05
6p-2	37.05	58.65	MTCH1, FGD2, PIM1, RNF8, GLO1, GLP1R, NFYA, NCR2, FOXP4, TFEB, PGC, PRICKLE4, BYSL, CCND3, **TRERF1, PRPH2**, TBCC, PTCRA, GNMT, PEX6, PPP2R5D, CUL7, PTK7, SRF AL133375.25, TTBK1, ZNF318, ABCC10, POLH, MAD2L1BP, VEGF, SLC29A1, HSP90AB1, NFKBIE, CDC5L, SUPT3H, RUNX2, DSCR1L1, MEP1A, TNFRSF21, CRISP2, CRISP3, PGK2, TFAP2B, IL17A, IL17F, MCM3, PAQR8 EFHC1, TRAM2, GSTA2, GSTA1, GSTA3, GSTA4, ICK, **GCLC**, BMP5, DST, ZNF451, BAG2, RAB23, PRIM2	42.60, 53.85	*36.65*
6q-1	90.35	90.75	no genes in region	*86.50*	*91.00*
6q-2	105.20	112.30	HACE1, LIN28B, BVES, PRDM1, AIM1, **QRSL1**, **PDSS2,** SEC63, SNX3, FOXO3, SESN1, CD164, SLC22A16, CDC2L6, AMD1, REV3L, TRAF3IP2, FYN	107.15	107.75
6q-3	115.00	120.40	FRK, SART2, FAM26F, KPNA5, ROS1, DCBLD1, GOPC, ASF1A	no peak in region	
6q-4	123.55	138.75	TCBA1, **TPD52L1**, HEY2, ESNA1, C6orf173, RNF146, PTPRK, ARHGAP18, AKAP7, ARG1, CRSP3, ENPP3, ENPP1, CTGF, RPS12, TCF21, SGK1, **MYB**, BCLAF1, MAP7, MAP3K5, IFNGR1, TNFAIP3, PERP, KIAA1244	125.55	135.60
7q-1	132.80	139.40	EXOC4, AKR1B1, AKR1B10, BPGM, **CALD1**, NUP205, PTN, DGKI, CREB3L2, TRIM24, KIAA1549, HIPK2, TBXAS1	134.40	*130.25*
7q-2	155.15	157.65	SHH, RNF32, MNX1, **UBE3C**, **DNAJB6**, PTPRN2	156.95	157.65
8q	127.40	132.95	FAM84B, MYC, GSDMC, ASAP1, ADCY8,	*120.95*	*121.95, 135.3*
10p-1	1.30	12.45	PFKP, COPEB, AKR1CL2, AKR1C1, AKR1C2, **AKR1C3**, NET1, GDI2, FBXO18, IL15RA, IL2RA, RBM17, PFKFB3, **PRKCQ**, ITIH5, ITIH2, KIN, GATA3, CUGBP2	5.10	6.45
10p-2	25.65	30.70	MYO3A, GAD2, APBB1IP, SSH3BP, MASTL, RAB18, **BAMBI,** MAP3K8	*24.90*	29.20
12p	0.25	11.60	**JARID1A**, B4GALNT3, RAD52, ERC1, WNT5B, ADIPOR2, FKBP4, FOXM1, TULP3, CCND2, FGF23, FGF6, RAD51AP1, DYRK4, AKAP3, KCNA6, KCNA1, KCNA5, NTF3, VWF, CD9, TNFRSF1A, SCNN1A, LTBR CD27, GAPDH, IFFO1, CHD4, ACRBP, ING4, ZNF384, COPS7A, MLF2, LAG3, CD4, GNB3, USP5, TPI1, ENO2, PTPN6, EMG1, LPCAT3, RBP5, PEX5, CD163, APOBEC1, GDF3, DPPA3 NANOGP8, SLC2A3, FOXJ2, ZNF705A, AICDA, M6PR, KLRG1, KLRB1, CD69, CLEC12A, CLEC1B, CLEC7A, AC022075.29, KLRC4, KLRC3, KLRC2, KLRK1, STYK1, CSDA, PRH1	0.25, *16.10*	0.25
13q	101.25	107.00	FGF14, ERCC5, SLC10A2, EFNB2	*99.55*	*110.35*
19q	36.50	41.75	ZNF507, PDCD5, C19orf40, CEBPA, CEBPG, PEPD, **GPI**, WTIP, HPN, FXYD3, FXYD5, LSR, USF2, HAMP, CD22, FFAR1, GAPDHS, ETV2, COX6B1, UPK1A, AD000671.1, PSENEN, HSPB6, APLP1 **HCST**, C19orf46, COX7A1, ZNF146	39.15	41.10
**Losses**					
3p	53.00	53.25	PRKCD	*53.85*	*62.55*
4p	15.65	27.05	QDPR, **MED28**, SLIT2, KCNIP4, PPARGC1A, SOD3, PI4K2B, RBPSUH, CCKAR	18.55	*11.00,* 26.65
5q-1	50.05	146.95	ITGA1, ITGA2, FST, HSPB3, ESM1, CCNO, DHX29, PPAP2A, DDX4, IL31RA, IL6ST, MAP3K1, **PLK2,** RAB3C, ERCC8, NDUFAF2, IPO11, SDCCAG10, ADAMTS6, ERBB2IP, CD180, PIK3R1, CCNB1, CENPH CDK7, TAF9, RAD17, OCLN, **NAIP,** **GTF2H2,** MAP1B, TNPO1, BTF3, ENC1, POLK, IQGAP2, F2R, F2RL1, S100Z, AC020898.6, THBS4, MSH3, RASGRF2, CKMT2, SSBP2, XRCC4, CSPG2, EDIL3 RASA1, **CCNH, NR2F1**, RFESD, RHOBTB3, GLRX, ELL2, PCSK1, ARTS-1, LRAP, LNPEP, RIOK2, CHD1, ST8SIA4, **PAM**, EFNA5, **FER,** CAMK4, STARD4, C5orf13, APC, MCC, TRIM36, **PGGT1B**, LOX, CSNK1G3, ZNF608, ALDH7A1, SLC12A2, ADAMTS19, RAPGEF6, ACSL6, IL3, CSF2, PDLIM4, IRF1, IL5, RAD50, IL13, IL4, 38230, GDF9, AFF4, HSPA4, TCF7, SKP1A, PPP2CA **UBE2B**, CAMLG, DDX46, PITX1, CXCL14, IL9, TGFBI, SMAD5, **SPOCK1,** WNT8A, KIF20A, CDC23, CDC25C, JMJD1B, EGR1, ETF1, HSPA9B, CTNNA1, SLC23A1, DNAJC18, UBE2D2, PSD2, NRG2, PURA HBEGF, EIF4EBP3, SRA1, CD14, DND1, HARS, PCDHB1, TAF7, PCDHGA6, PCDHGC5, DIAPH1, HDAC3, FCHSD1, PCDH1, RNF14, SPRY4, FGF1, ARHGAP26, NR3C1, HMHB1, YIPF5, POU4F3, PPP2R2B, DPYSL3	57.70, 70.75, 89.75,102.30, 116.25, 133.80	70.05, 89.85, 108.60, 118.55, 136.30
5q-2	161.40	171.20	CCNG1, HMMR, MAT2B, WWC1, DOCK2, FOXI1, KCNMB1, GABRP, RANBP17, TLX3, NPM1, FGF18	*161.05, 178.30*	*157.30*
10q-1	80.65	95.50	SFTPA1, SFTPD, **NRG3**, GHITM, RGR, GRID1, WAPAL, BMPR1A, SNCG, MINPP1, PTEN, LIPK, ACTA2, FAS, **IFIT2**, **IFIT3**, ANKRD1, PCGF5, HECTD2, PPP1R3C, TNKS2, BTAF1, 36950, IDE KIF11, HHEX, MYOF, CEP55	83.30, 90.15	91.05
10q-2	105.55	111.35	SLK, COL17A1, GSTO1, GSTO2	109.70	108.60
12q-1	47.70	48.35	RHEBL1, TUBA1B, TUBA1A, DNAJC22	no peak in region	*42.15*
12q-2	54.30	59.25	LACRT, DCD, MUCL1, ITGA7, BLOC1S1, CD63, GDF11, AC073487.34, DNAJC14, MMP19, DGKA, SILV, CDK2, RAB5B, ERBB3, PA2G4, SMARCC2, RNF41, OBFC2B, CS, IL23A, STAT2, TIMELESS, **GLS2, PTGES3**, NACA, RDH16, ZBTB39, MYO1A, STAT6, LRP1, INHBC, GLI1, ARHGAP9, DDIT3, PIP4K2C, SLC26A10, B4GALNT1, AC025165.27, CENTG1, TSPAN31, CDK4, CYP27B1, FAM119B, CTDSP2, XRCC6BP1, LRIG3, **SLC16A7**	55.25	58.40
14q-1	38.30	44.75	SIP1, PNN, MIA2, CTAGE, **LRFN5**, FANCM	40.65	*36.35*
14q-2	48.35	92.95	RPS29, PPIL5, POLE2, SDCCAG1, ARF6, SOS2, CDKL1, SAV1, NIN, GNG2, C14orf166, NID2, PTGER2, ERO1L, BMP4, CDKN3, CGRRF1, GCH1, MAPK1IP1L, LGALS3, DLG7, FBXO34, **KTN1**, PELI2 OTX2, **ARID4A**, TIMM9, DACT1, RTN1, PPM1A, SIX1, MNAT1, PRKCH, HIF1A, KCNH5, RHOJ, PPP2R5E, ESR2, AKAP5, HSPA2, SPTB, GPX2, RAB15, MAX, **FUT8**, GPHN, MPP5, EIF2S1 PIGH, ARG2, RDH11, RAD51L1, ZFP36L1, ACTN1, WDR22, ERH, SLC10A1, ADAM20, MAP3K9, PCNX, RGS6, PSEN1, NUMB, HEATR4, PNMA1, ENTPD5, LTBP2, RPS6KL1, PGF, MLH3, ACYP1 NEK9, FOS, JDP2, BATF, FLVCR2, TGFB3, ESRRB, VASH1, GSTZ1, AHSA1, ALKBH1, SNW1, ADCK1, **DIO2**, TSHR, **SEL1L**, GALC, GPR65, SPATA7, PTPN21, FOXN3, TDP1, AL512791.3, RPS6KA5 GPR68, FBLN5, TRIP11, ATXN3, RIN3, LGMN, GOLGA5, CHGA, MOAP1, BTBD7, COX8C	57.35, 79.90 *98.00*	55.20, 66.15, 81.0, *95.05*
15q	35.10	49.65	SPRED1, THBS1, EIF2AK4, BMF, BUB1B, PAK6, PLCB2, CASC5, RAD51, FAM82C, DNAJC17, PPP1R14D, SPINT1, RHOV, DLL4, INOC1, ITPKA, LTK, TYRO3, MAPKBP1, VPS39, CAPN3, TTBK2, CCNDBP1 LCMT2, TP53BP1, CKMT1B, PDIA3, WDR76, CASC4, **B2M**, DUOX2, DUOXA2, DUOXA1, DUOX1, C15orf48, SLC30A4, **C15orf21**, DUT, FBN1, COPS2, FGF7, DTWD1, HDC, USP8, TRPM7, TNFAIP8L3, CYP19A1 GLDN	44.40	*33.70,* 42.85

Table 2b. Overlapping differential CNAs of luminal breast tumors vs *BRCA1*-mutated breast tumors and BLBCs

Chr - region	Start (Mb)	End (Mb)	Cancer related genes in regions of overlap<br>bold: peak locations of KSE curves of luminal breast cancers	peaks (Mb)<br>luminal
**Gains**				
1q	176.70	215.40	RALGPS2, ANGPTL1, **ABL2**, SOAT1, CEP350, LHX4, XPR1, IER5, GLUL, RGSL1, RNASEL, RGS16, RGS8, DHX9, LAMC1, LAMC2, NCF2, RGL1, FAM129A, RNF2, TPR, PTGS2, PLA2G4A, FAM5C RGS18, RGS1, RGS13, RGS2, UCHL5, TROVE2, GLRX2, CDC73, CFH, ASPM, NEK7, PTPRC, NR5A2, KIF14, PKP1, CSRP1, TIMM17A, RNPEP, ELF3, PTPN7, UBE2T, PPP1R12B, JARID1B, RABIF ADIPOR1, PPFIA4, MYOG, CHI3L1, CHIT1, BTG2, FMOD, OPTC, REN, KISS1, PLEKHA6, PPP1R15B, **PIK3C2B, MDM4**, LRRN2, CNTN2, RBBP5, RIPK5, NUAK2, LEMD1, PCTK3, ELK4, SLC45A3 RAB7L1, CTSE, SRGAP2, IKBKE, RASSF5, DYRK3, MAPKAPK2, IL10, IL20, IL24, FAIM3, PIGR, C4BPA, CD55, CR1, CD46, CD34, CAMK1G, LAMB3, G0S2, HSD11B1, HHAT, KCNH1, RCOR3 TRAF5, NEK2, DTL, PPP2R5A, ATF3, FLVCR1, RPS6KC1, PROX1, SMYD2, PTPN14, CENPF, KCTD3, ESRRG	177.35, 202.75
8p	35.95	38.90	GPR124, ADRB3, EIF4EBP1, ASH2L, STAR, LSM1, BAG4, PPAPDC1B, WHSC1L1, FGFR1, TACC1	*41.5*
16p	4.30	27.75	CORO7, DNAJA3, HMOX2, C16orf5, PPL, USP7, GRIN2A, FAM18A, CIITA, SOCS1, PRM2, LITAF, GSPT1, TNFRSF17, ERCC4, MKL2, BFAR, PLA2G10, PDXDC1, RRN3, **MYH1**1, ABCC1, ABCC6, AC138811.3 COQ7, C16orf62, DCUN1D3, ANKS4B, EEF2K, CDR2, HS3ST2, SCNN1B, PALB2, PLK1, ERN2, AC130454.2, PRKCB1, RBBP6, ARHGAP17, AQP8, NSMCE1	15.8
**Losses**				
6q-1	79.05	87.25	HMGN3, TTK, **FAM46A**, TPBG, TBX18, NT5E	82.35
6q-2	142.75	149.25	AIG1, PEX3, PLAGL1, UTRN, EPM2A, FBXO30, SHPRH, GRM1, RAB32, SASH	no peak in region
6q-3	156.90	157.85	no genes	*160.05*
11q	104.45	125.35	AASDHPPT, ALKBH8, RAB39, CUL5, ACAT1, ATM, DDX10, ARHGAP20, POU2AF1, BTG4, SNF1LK2, PPP2R1B, CRYAB, HSPB2, DLAT, TIMM8B, SDHD, **TTC12**, DRD2, ZW10, USP28, HTR3A, ZBTB16, NNMT REXO2, CADM1, BUD13, APOA1, PAFAH1B2, TAGLN, PCSK7, IL10RA, TMPRSS4, CD3E, UBE4A, MLL, ARCN1, DDX6, CXCR5, BCL9L, FOXR1, SLC37A4, HYOU1, H2AFX, MIZF, CBL, MCAM, RNF26 C1QTNF5, USP2, THY1, PVRL1, TRIM29, POU2F3, ARHGEF12, UBASH3B, CRTAM, HSPA8, SCN3B, VWA5A, TBRG1, SPA17, ROBO4, HEPACAM, FEZ1, EI24, CHEK1, DDX25	112.30, *126.60*
13q	91.70	95.50	DCT, ABCC4, CLDN10, DNAJC3	*99.1*
16q	45.15	88.50	ORC6L, DNAJA2, ABCC11, SIAH, ADCY7, BRD7, CARD15, CYLD, **RBL2**, IRX5, MMP2, SLC6A2, CES1, GNAO1, AMFR, MT3, MT2A, MT1A, MT1F, MT1G, MT1H, NUP93, SLC12A3, CCL22 CX3CL1, CCL17, CIAPIN1, GPR56, KATNB1, MMP15, CSNK2A2, NDRG4, CDH8, CDH11, CDH5, TK2, CDH16, RRAD, CES2, CBFB, TRADD, NOL3, E2F4, HSD11B2, CTCF, ACD, PSKH1, PSMB10 LCAT, SLC12A4, DDX28, NFATC3, SMPD3, CDH3, CDH1, HAS3, AC009027.10, PDF, TERF2, NFAT5, NQO1, WWP2, AARS, DDX19B, DDX19A, FUK, SF3B3, VAC14, HYDIN, CALB2, ZNF23 CHST4, TAT, ZNF821, DHODH, HP, DHX38, ATBF1, PSMD7, GLG1, MLKL, FA2H, BCAR1, CFDP1, TERF2IP, ADAMTS18, WWOX, MAF, DYNLRB2, **ATMIN**, PLCG2, HSD17B2, CDH13, OSGIN1, MBTPS1 WFDC1, COTL1, COX4I1, IRF8, FOXF1, FOXC2, FOXL1, FBXO31, SLC7A5, IL17C, CYBA, RNF166, FAM38A, CDT1, APRT, CBFA2T3, CDH15, ANKRD11, SPG7, RPL13, CPNE7, DPEP1, CDK10, FANCA TCF25	52.30, 79.40
